# Clients’ Perspectives Regarding Peer Support Providers’ Roles and Support for Client Access to and Use of Publicly Funded Mental Health Programs Serving Transition-Age Youth in Two Southern California Counties

**DOI:** 10.1007/s11414-022-09792-6

**Published:** 2022-03-02

**Authors:** Sarah Hiller-Venegas, Todd P. Gilmer, Nev Jones, Michelle R. Munson, Victoria D. Ojeda

**Affiliations:** 1 Herbert Wertheim School of Public Health, University of California, 9500 Gilman Drive, CA 92093-0725 La Jolla, USA; 2 School of Social Work, University of Pittsburgh, 2117 Cathedral of Learning, 4200 Fifth Ave., Pittsburgh, PA 15260 USA; 3 Silver School of Social Work, New York University, New York, NY USA; 4 School of Medicine, University of California, CA 92093 La Jolla, USA

**Keywords:** mental health services, transition age youth, peer providers, recovery, role modelling, mentoring, social support

## Abstract

Peer providers are increasingly used by mental health programs to engage transition age youth (TAY, age 16-24) living with serious mental illness. This study elicited TAY clients’ perspectives on peer providers’ roles, responsibilities, and contribution to TAYs’ use of mental health services. In 2019, six focus groups were conducted with TAY clients (n=24) receiving publicly funded mental health services in Southern California. Results from this analysis included four themes that illustrated the role of peers as perceived by TAY clients, including: 1) building client–peer provider relationships, 2) engaging with mental health services, 3) role-modelling recovery and supporting skill acquisition to instill hope and empowerment, and 4) peer roles and experiences specific to racial/ethnic concordance. These findings provide needed perspectives on the evolving role of peer providers in mental health services programming for TAY clients.

## Introduction

Transition age youth (TAY, ages 16-24) with serious mental illness (SMI) face exceptional challenges as they move from adolescence to adulthood, including service barriers related to transitioning from child to adult systems of care and for some, the loss of financial and social support resulting from emancipation from foster care.^1–4^ Approximately 75% of SMI, including schizophrenia-spectrum disorders, severe bipolar disorder, and severe major depression, manifest by age 24.^5^ In 2019, the prevalence of SMI in the USA was highest among young adults age 18-25 at 8.6%, compared to 6.8% for ages 25-49 and 2.9% for age 50 or older.^6^ Despite the early onset and higher prevalence of mental illness among TAY, only 56.4% of young adults with SMI received mental health services, compared to 65.1% among ages 25-49 and 74.3% for age 50 or older. ^6^ Across all adult age groups, African-Americans and Latinos with mental illness are less likely to receive mental health care than their non-Hispanic white counterparts; these patterns are especially marked among African-American and Latino TAY.^7^

Peer support services are delivered by individuals with lived experience of mental illness who assist others in their recovery by providing emotional support (i.e. showing care or sympathy, understanding, listening), instrumental support (i.e. assisting with tangible needs, such as material goods, money, or providing services like transportation), informational support (i.e. providing advice or specialized knowledge about a specific topic), and appraisal support (i.e. help making decisions, acting as a sounding board, providing feedback).^8,9^ Peer support services may improve youth engagement by offering mutuality, empathy, hope, and trust,^10^ and reducing social isolation, disempowerment, and frustration.^10,11^ Peers may also serve as role models due to their lived experienced and achievement of personal or professional goals.^12,13^ This relationship may enhance a client’s connection to other mental health providers. Peer providers may also help engage clients have previously been stigmatized by mental health service providers.^10^ Employing peers who share clients’ cultural backgrounds may also help reduce disparities in access to and engagement in mental health services by providing culturally and developmentally appropriate supports.^5,11,14^

The use of peer support in mental health service systems has increased worldwide since the 1990s.^11,15,16^ Yet, systematic reviews and meta-analyses of the use of peer support among adults reach differing conclusions due to variations in the study design, participants recruited, and outcomes assessed.^17–19^ Challenges with evaluating the effectiveness of peer support include the diverse array of roles and responsibilities adopted by peer providers, particularly in naturalistic community-based settings and lack of consensus^20,21^ and consistency in the operationalizations of peer roles.^22,23^ Notably, qualitative studies on peer support often focus on the perspective of peer providers rather than clients.^16,19^ Additionally, extant literature does not disaggregate the experiences of TAY clients which challenges the field to move forward in understanding the impact of peers as an intervention for TAY living with SMI.^24,25^

This study reports qualitative findings from a sequential mixed-methods study. Quantitative analyses based on administrative data first found that peer support was associated with increased use of outpatient mental health services and greater use of services in programs.^26,27^ This qualitative investigation thus sought to expand upon quantitative findings and it examines TAY client perceptions of peer providers as it relates to support provided to facilitate their recovery and well-being.

## Methods

### Study setting, participants, and data collection

The study was conducted among in San Diego and Los Angeles Counties; both counties provided the investigators with a list of county-funded mental health programs that serve TAY. In 2018-2019, these programs were queried to determine their use of peer providers. Purposeful sampling was used to identify the six programs in which peers provided a range of services to a diverse population of TAY.^27^ Programs were provided with a recruitment flyer and suggested messaging to recruit TAY clients to join focus groups. Focus groups were conducted between September and November 2019. To achieve equal representation in each county, the principal investigator conducted six client focus groups (n=3 groups in Los Angeles; average number of participants: n=5.6, n=3 groups in San Diego, average number of participants: n=2.3) with 24 participants (n=17 males, n=7 females); other demographic data were not collected. Participant recruitment was terminated due to saturation of themes (i.e. no new data were collected with additional participants)^28^ with the existing sample. Each focus group lasted between 25 and 60 minutes, with an average duration of 37 minutes; they were digitally recorded. Refreshments and $20 gift cards were provided to participants. Participants provided their written informed consent to join the study.

### Focus group interview guide

To investigate TAY clients’ perceptions of peer providers as it relates to support provided to facilitate their recovery and well-being, the interview guide elicited information about 1) the activities that TAY clients engaged in with peer providers, their relationship with their peer providers, and how their peer provider compares with clinical staff, 2) what characteristics or skills clients desired in a peer provider, 3) how interacting with a peer provider influenced their experience using mental health services, and 4) how peer providers may have influenced clients’ way of thinking about mental health and recovery. Before initiating the discussion, the facilitator first defined a peer as “someone who has lived experience with mental health challenges and assists others in their recovery”.

### Data management and analysis

Focus group audio files were transcribed verbatim by study staff. Transcripts were coded and analyzed using MaxQDA 2020 software using a combined deductive and inductive approach to thematic analysis.^29^ A preliminary coding scheme was developed based on the interview guide; emergent codes were identified and added. The team layered intersectional codes to cross-reference segments of text representing a specific topics, qualities, or characteristics found throughout the focus group across different topics (e.g. Latino TAY-Specific”).^30^ The coding scheme was applied to two transcripts by four study team members (VO, SH, and two research assistants) to identify any additional emergent codes and further refine the structure of the coding scheme. Revisions to the coding scheme were based on group discussion and consensus, and it was applied to another two transcripts by SH and another coder to assess inter-rater reliability. After resolution of conflicts in how codes were applied, satisfactory inter-rater reliability was achieved (i.e. 81%), and the remaining four transcripts were coded.^31–33^

Coded text segments were collated into code reports for further analysis. Within these code reports, the analysts summarized each code across all the focus groups to identify instances of agreement and disagreement, and also categorized any variations across thematic areas to develop subthemes. Axial coding^34,35^ was used to categorize subcodes and themes into concepts aligning with those found in existing literature on peer and social support to reflect the roles of peer providers and the mechanisms by which peers are thought to influence the experiences of clients in mental health services. Code frequencies by participant and focus group were generated to help the authors prioritize concepts and themes. Overlapping codes (i.e. the same phrase being coded with multiple codes) were examined to facilitate the process of identifying themes and subthemes.

### IRB statement

This study was approved by the Human Subjects Research Protections Program at the University of California, San Diego, Los Angeles Department of Mental Health, and San Diego County Department of Behavioral Health Services in accordance with the Privacy Rule of the Health Insurance Portability and Accountability Act of 1996. The study included a decisional capacity assessment that was completed by the facilitator before initiating the interview, and no individuals were excluded from the study per this protocol.

## Results

A total of four themes were identified that embodied the role of peers as described by TAY participants, each with several subthemes, as follows: 1) building client–peer provider relationships, 2) bridging and engaging with mental health services, 3) role-modelling recovery and supporting skill acquisition to instill hope and empowerment, and 4) peer provider roles and experiences specific to young adults, gender, and racial/ethnic minority groups.

### Theme 1: Building client–peer provider relationships

We identified three subthemes related to the relationships that were built between clients and peer providers including “building trust and connection,” “addressing basic needs and goals,” and “providing emotional support.” Table 1 provides illustrative participant quotes for these subthemes.

**Table 1.** Building Client–Peer Provider Relationships

Subtheme	Exemplary Quotes
**Building Trust & Connection**	**RELATABLE/LIVED EXPERIENCE** *If they haven’t been through the same thing as you have, then you don't know where they're coming from, they don’t know where you’re coming from.* (Male 1, Group A) *The fact that I feel like she [my peer] knows where I'm coming from. I’m not saying anything bad about my therapist, but they just have this feeling that they might not know completely know what I go through... But I feel like she basically knows where I'm coming from, she's in my scene now. Like, the therapist, I feel like they have… not power over me, but coming more from a spectator look.* (Male 1, Group B) **SUPPORTIVE** *Honestly, they’ve always really just been supportive through everything I wanted to do and at the end of the day I'll come back and tell them and they'll be like ‘oh yeah you should do this and that and that,’ and be supportive … one time when I had to remember this whole speech, it was… something I had to remember for a job… I was here and I was really stressed out because I couldn't remember it and they were being really supportive, it was like… ‘here’s a room to go to, and just calm down, we’ll get you some water,’ and I was like I can't do it, but they were really supportive the whole time I learned how to do it.* (Female 2, Group A) **TRUST** *I feel like for a peer support, like opening up and telling stories of what happened to them, like relating to people, I think that'd be a really good, an important step for peer support. I think I should be a mandatory thing, in a sense. Because it brings up a level of trust and it will make people feel more comfortable to talk to them. I feel like that should be something important.* (Female 1, Group A) *A lot of people don't like being asked questions but when it comes from more comfortable people like from [the peers], it comes in handy to actually get to understand somebody. Me personally, there's a certain boundary of trust I have to be able to put in somebody before I just answer questions left and right.* (Male 2, Group A)
**Addressing Basic Needs & Goals**	**BASIC NEEDS- TRANSPORTATION, HOUSING, SHOPPING, HYGIENE, ETC.** *And they provide transportation. Man, [my peer provider], he’ll drive you wherever you want, swear to god, he would, took me all the way to [neighboring city], from here, it was raining.* (Male 1, Group A) *He's helped me a lot with shopping, hygiene, he's a pretty cool dude… And transportation… when I got out of jail, I didn't have shoes and he got me everything I needed, toothpaste, body wash, clothes.* (Male 1, Group D) *They helped me get out of [being] homeless.* (Male 2, Group A) ***FLEXIBILITY/KNOWLEDGEABLE ABOUT RESOURCES*** *They’re very flexible… whenever you ask for something, they’re always like ‘yeah, we can do that, we can do this.’ They’re always helping us with the showers… They're just very helpful. They have very good suggestions too.* (Male 5, Group E) *They definitely help point you to other resources, like say you need something or are struggling with something, they obviously point you in the direction of whatever else that [the program] offers. (Female 1, Group C)* **IMMEDIATE GOALS** *Like [my therapist is] a great person but with her, it was more of a serious thing, like we're here to work, we're here to fix whatever you have and whatever your goals are. And then with peer support, they do that, like they support our goals and everything but with them it's a different kind of goal, like, what kind of goals do we have realistically, like hey, what do we need, do we need a job, what steps do you do.* (Male 3, Group C)
**Providing emotional support**	**LISTENING/HAVING SOMEONE TO TALK TO** *And I can talk to [my peer] about anything, everything that's very confidential. I met her through one of our groups, so I felt like ‘okay I'm not the only one dealing with what I'm going through.’ Because at first, you really don't know, you think you're out there by yourself, but once you get to the program that you’re in and deal with peer counselors or whatever, you find out that there's other people just like you and you might not be on the same level but everyone is different and everyone is loving so it's like a big family.* (Female 1, Group F) ***Female 1:*** *Someone who understands like everything you're coming from, who will listen. Especially listens because, man, it means a lot to a lot of people if somebody like really listens to you.* ***Female 2:*** *I agree.* ***Male 1:*** *Really understands where you're coming from.* ***Male 2:*** *Well, it’s like solving a math equation, if you don’t understand it you can't properly solve it.* (Multiple Participants, Group A) **SUPPORT WITHOUT PRESSURE** *I’ve seen how it is important that not only we connect on a human level but just being able to… come to places where you feel safe, you don’t feel judged, you don’t feel the pressure. That’s one thing I love about this place, you work at your own pace, nobody’s really pushing you, it is self-reliant, and it’s about you showing up.* (Female 3, Group F) *There was [another peer provider] used to be here… [she] helped me to recognize that I could recover and be healthy. I mean, it’s not, how can I put it, it's not what she said, it was the way she was saying it… Like she wouldn’t say, ‘you need to do this,’… she said ‘oh, you should come to the computer room,’ and I said okay. She didn't say you should do any of the work… so I would just go in there and dang, just sat around, and then one day I just said you know, I want to start doing some of the work, on my own… the next thing I know I was volunteering and being here, you know. So it’s like, the person who can encourage you, you know, the person who encourages you and remembers where they came from.* (Male 3, Group F)

#### Building trust and connection

Clients described their interactions with peers, how they related to peer providers, how peers compare to clinical mental health staff, and what they felt were the most important qualities and characteristics of peers. Compared to clinicians, peers were perceived to be less formal and more understanding or less judgmental. Participants described communication with their peer as easy, comfortable, or “like talking to family”. The importance of relatability and understanding generated through peers’ lived experience was described consistently across all focus groups. Participants described specific ways in which peers were especially supportive or dependable: peers were described as going above and beyond to provide help in times of need. Examples provided by clients included peers’ helping clients prepare to give a speech or helping the client seek care for a physical injury.

#### Addressing basic needs and goals

Clients described ways in which peer providers helped them with basic needs through direct services or referrals to other services or community resources. For example, peers addressed basic needs and helped clients with grocery shopping, assisted with personal hygiene, identified housing, or provided transportation. Clients also indicated that peers offered information and linkages to other services and resources, either within the organization or in the broader community. Clients emphasized that peers had the flexibility and knowledge to address their multiple and diverse needs. One participant specifically noted that peers helped them with their immediate or “real life” goals such as getting a job—processes that were distinct from those of therapists who have a narrower focus on longer-term or mental health-specific goals.

#### Providing emotional support

The majority of clients explicitly described peers’ provision of emotional support. When asked about key qualities in a peer, clients reported that peers should be friendly, empathetic or non-judgmental, and a good listener. Clients appreciated that peers were supportive without pressuring clients to do anything they did not want to do. Clients felt that this low-pressure approach facilitated engagement with services or helped them set or achieve recovery-related goals such as going to school, working, or engaging in social activities.

### Theme 2: Bridging and engaging with mental health services

Clients reported that peer providers helped them access mental health services, often by leveraging the client–peer relationship. This analysis resulted in the identification of three sub-themes for peer support for engaging with mental health services, which included “reducing barriers to mental health services,” “collaborating with mental health team,” and “building mental health literacy to navigate mental health services” (see Table 2 for illustrative quotes).

**Table 2.** Bridging and Engaging with Mental Health Services

Subthemes	Exemplary Quotes
**Reducing Barriers to Mental Health Services**	**REDUCING BARRIERS** *[Peers] provided a lot of things for me, which I never knew there's stuff like this that’s provided. Because if it wasn’t for them, my life would be much worse, and they make sure that where I stay is comfortable for me and provide enough food and services that I needed… My therapist, I didn’t have a therapist for a while, but now I do. But it's okay, because there’s a service pretty often, and they make sure you get there on time. Sometimes they pick you up and everything so.* (Male 2, Group D) **AMENITIES DRAW CLIENTS IN** ***Male 2:*** *They even have a little snack store or whatever for people that want to use their stamp cards and the stamp card just helps you get whatever you want from there.* ***Facilitator:*** *What you have to do to earn the stamps ?* ***Male 1:*** *Go to Class* ***Male 2:*** *yeah you go to class, or, they call it groups…* ***Facilitator:*** *Is that the ones on the board that I saw, like the meditation, anger management? …****[group agreement]*** *Ok, cool. so, you guys enjoy that?* ***[group agreement]*** (Multiple participants, Group E) *All you have to do is call [the peer outreach team] and tell them where you are, give them your name and destination… your name, destination, and where you are, and they’ll pick you up and drop you off. But you do the classes in order to get a ride, to get back where you were.* (Male 1, Group E) ***Facilitator:*** *Was it the outreach team that made you familiar with the program?* ***Male 4:*** *No, it was knowing that I could come here during the day and shower and, you know, have a meal, it's a cool thing. I was actually going to try to go talk to a therapist [soon], because like, emotionally I wasn't there. So, I need to talk to somebody.* (Male 4, Group E)
**Collaborating with Mental Health Team**	*But [the peer provider will] get notes from the therapist. Our therapist will be like ‘oh, I’m going to tell [your peer] to help you with this service, this service or this or this’ or give a note and [your peer] would later check up on you, like ‘oh I heard you need this’ and they’ll just update if you need to know more or not, so they’ll just help you out with services that your therapist mentioned that [the therapist] can’t help you out with but that your peer support could.* (Male 3, Group C) *They [peer providers] have good cooperation skills…. With us… and with each other. They help each other to help us too.* (Male 3, Group E) ***Facilitator:*** *And is there something different about the support that you get from the peer partner versus the therapists or the case manager?* ***Male 1****: It’s like the same thing, honestly.* ***Male 2****: Yeah. They give each other connections basically. And our connection with them. So basically, if something is wrong with say, the client, they would tell it…* ***Male 1****: address it. Not to you, but…* ***Male 2****: To each other and then they would go to you and tell you whatever.* ***Facilitator:*** *So, they work in a team, your peer partner works with the therapist or your case manager…* ***Male 2****: Yeah so, it's like, that team, whatever their name is, it’s that team.* ***Facilitator:*** *Has that been your guys' experience too? That there’s communication between your peer partner and other people here?* ***Male 4****: Yeah. That's been my experience.* (Multiple Participants, Group E)
**Building Mental Health Literacy to Navigate Mental Health Services**	***Facilitator:*** *Do [peer providers] make it easier to use the mental health services?* ***Male 3:*** *To be honest, yes. When I was here I was trying meds for a bit, so you basically add another person to your team which is a doctor so now you have three people which is more work* ***Facilitator:*** *Ok so it would be the therapist, the peer, and now the doctor.* ***Male 3:*** *Yes, and so we're just going off, you know, diagnosis and everything and you know sometimes when it comes to the medical field or like anything you choose, you have to click with the person. If you don't, you just- we ain't vibing, sorry. Deuces. So like with [my peer] it was helpful like ‘Hey how do I tell this person that we're not vibing or we’re not, like you know.’ You can just tell them like, oh, they said something to me, which was always helpful, trying to have someone work it out for you or help you like oh, well you could do this and this or you can take these steps, so that was helpful for peer support, they will just be by your side, like help you out, even with the news and everything you hear.* (Male 3, Group C) *I'll update [my peer] or she’ll ask, like ‘how was your appointment, how do you feel,’ that was more the thing about the peer support is more like, just checking up on you, you know, ‘how you're feeling, are you cool, I heard you had a pretty intensive session last week, are you okay?’ So that was that's how they help, they’re more like little ice packs, and you go out on the field and then bam, you got an ice pack.* (Male 3, Group C)

#### Reducing barriers to mental health services

Peer providers were perceived to change clients’ relationship to mental health services by reducing barriers to accessing services or facilitating linkages to services. Specific examples included providing information about mental health services, setting up appointments with providers, or providing transportation to services. Several clients described scenarios in which amenities or services managed by peers, such as showers or laundry, initially drew them into the mental health services program; clients were later persuaded to engage further in service use by agreeing to meet with a therapist or attend a group session. Peers in one program provided “stamps cards” for attending group sessions which clients could trade in for goods at an onsite convenience store. Clients were generally appreciative of these exchanges, and importantly, no clients expressed feeling coerced or compelled to participate.

#### Collaborating with mental health team

Nearly half of the clients interviewed indicated that their peer providers communicated with their mental health team of clinicians, other peers, or other program staff regarding their needs for referrals to additional services.

#### Building mental health literacy to navigate mental health services

Several clients described instances whereby peer providers provided specialized informational support that helped build their understanding of how to navigate mental health services. Several clients specifically reported that their peers helped them navigate shared decision making and communication of any concerns with taking medication for their mental health issues to the treatment team. In one example, a client described how a peer helped him understand that if he felt that a member of his mental health team was not a good fit for him (in this example, his psychiatrist), he could simply explain that and ask for someone else. This client also described how he and his peer would discuss how his therapy sessions went, and how she helped him cope when therapy was especially intense, comparing her to an ice-pack for an athlete after they get off the playing field.

### Theme 3: Modelling recovery and supporting skill acquisition to instill hope and empowerment

Peers’ lived experienced was important to clients and increased their feeling of being understood and was a defining feature distinguishing peers from other program staff. Additionally, the third theme also included clients’ descriptions of how peers served as “recovery role models,” which gave them hope for the future, and helped with “building or practicing skills for recovery” (please refer to Table 3 for exemplary quotes).

**Table 3.** Modelling Recovery and Supporting Skill Acquisition to Instill Hope and Empowerment

Subtheme	Exemplary Quotes
**Recovery Role Models**	**FEELING LESS ALONE** *The main thing was [my peer] just relating to me, the whole thing was like, you know, having a new diagnosis… and feeling like you're alone. And her listening and telling me like ‘I know exactly what you mean and I know exactly how you feel and I've gone through that too during these certain phases of my life. I totally get that.’ So that's the biggest thing that I got from [my peer] was the feeling of not being alone.* (Female 1, Group C) *It’s kind of essential for you to interact with at least one other person who knows exactly what you're going through or has gone through what you're going through, so you don't feel like you're abnormal or weird or anything like that. So, I think it's essential for someone who really wants to recover.* (Female 1, Group C) **SEEING AN EXAMPLE OF SUCCESSFUL RECOVERY** *It's really helpful when you have a new diagnosis and especially when you're young, it’s peaking. Because your brain is still developing and it feels kind of like the end, but it's really refreshing to see someone who's actually a functioning adult with the same thing. So, sharing similar experiences.* (Female 1, Group C) *The education is great that they have it, but sometimes you need someone to say ‘this is how it is, I've been through this and this is how you can change, this is how I changed.’ So sometimes we need people to say ‘no, I've been through this too, and this is how you can make it better.’* (Female 4, Group F) *Everyone is struggling and everybody's on a different level and you can see people evolving and that's really wonderful because you know [recovery is] possible for you… You can see that it’s possible and you can set goals and I just… it's life skills, you know, it should be taught in grammar school.* (Female 2, Group F) **HOPE FOR THE FUTURE** *I've been inspired to go to the peer training program, so I did that, peer, what is it, the peer specialist program… it helped me because as I continue to get better and get well and walk in my wellness and complete the goals that I have, I can look at certain people that come up here as newb- I was gonna say newbies. But, they made me think of that's how I was when I first came, so that makes me gravitate to that person and try to be of assistance and to help them in any way.* (Male 3, Group F) *They helped me a lot, the peer partners. They helped me see a better view of life… they were just very positive. … I’m not going to name names, but some have been through the struggle. You know, and they just gave me an open eye that I could do it and life is worth it.* (Male 1, Group E) *To put it frankly, I just felt like she understood me. And basically that helped me to motivate myself to be better. She also said that I could… that I should give it a try to become a peer specialist also and that’s not for me, but it does give me some kind of hope that I can make it.* (Male 1, Group B)
**Building/ Practicing Skills for Recovery**	**INCREASING INDEPENDENCE IN EVERYDAY TASKS AND INTERACTIONS** *With [my peer], he gets me out of the house at first, and then… as the months progress, we focused more on being productive and what's stopping me from being productive… I have a problem staying motivated with doing tasks. [He] helped me focus on what would be the issue, if there was something else that was bothering me or just, I don't know, if it's just lacking energy or just if it was me… [he taught me strategies like] taking a break when you need to, not trying to take it all in one big bite, but trying to break it down.* (Male 2, Group C) *[My peer and I] started going to places because I have a big fear of getting outside my house. It's a bit embarrassing, but… I have this tremendous fear that people are looking at me… I have paranoia, psychosis. She takes me places, I'm learning a lot about it. Like last time we went to the mall… I actually don't want to be here [at this program] anymore, not that I don’t enjoy the services, but I want to seem normal and more stable… And that's why I'm pushing myself to go to different places with people now.* (Male 1, Group B) **REINFORCING MENTAL WELLNESS SKILLS DURING CHALLENGING TIMES/SETBACKS** *One of the coping skills was when I first came here [my peer] said I had a lot of stuff to let go, he said ‘let it go,’ that just encouraged me so much, I do t-shirts and things like that, so I’m going to make a t-shirt that says that. And then learning that we have no power over the outward environment, we have to be concerned about this environment right here, taking care of ourselves, and a lot of times, I know for myself I was more into taking care of everybody else and letting myself go, so that has helped me a great deal.* (Male 3, Group F) *When I broke my leg, I had to have surgery and I literally just backed up, like everything just sort of backed up, everything I learned stalled because I was just broken... And after that she told me ‘don't forget your teachings,’ like you know she just reminded me, ‘don’t forget what you learned, it's just a little bump’ and that's the thing, we, as young adults that we forget, that sometimes when something happens either we have simple ways of sitting down and shutting down or in my case I just cry about it and then get over it. But they remind us that there are other options… there’s always other ways to figure out a problem.* (Male 3, Group C) *When I started using drugs again, I started making excuses, not go to school, I have some anxiety. She said,* ***‘****well sometimes you gotta, you know, go through the consequences of your actions’ and that’s true, because that’s my own action that I put upon myself. And she just said, ‘sometimes you just have to, it’s scary, but you have to do it,’ just, you know. Since I don't take pills, like I was asked, but she told me ‘if you want to get better, the pills, taking pills on time and regularly on schedule can help me.’ So, I appreciate that.* (Male 2, Group D)

#### Recovery role models

Clients often associated the importance of peer providers’ lived experience with mental health issues to being inspired by the peers, who in turn gave them hope for the future. Peers were felt to help clients address lowered self-expectations or self-stigma and helped to bolster clients’ expectations for recovery. Several clients described ways in which peers inspired them, often through discussions about how they had learned to manage their mental health, pursued their goals and reached milestones; these discussions were substantiated by peer’s success in personal and professional domains. Clients described peers as credible guides who could keep them motivated and provide them with informational and emotional support to stay on their personal path to recovery. Specifically, clients described realizing they were not alone in their struggle due to meeting peers who had faced and overcome similar mental health challenges. Clients reported feeling more hopeful about being able to reach their personal goals, and a few indicated an interest in becoming peers themselves to help others as the peers had helped them.

#### Building/practicing skills for recovery

Many clients identified how the peer providers helped them build or practice skills that supported their recovery, including everyday skills such as dealing with social anxiety in public settings, increasing their independence, and reinforcing skills such as coping skills or anger management that were initially taught in a mental health services setting (e.g. in peer-led group sessions or in one-on-one therapy with clinicians). Peers encouraged clients to remember and apply those skills when faced with setbacks or challenges (e.g. relapsing on drugs, recovering from a physical injury, or experience a period of more intense mental health symptoms). Clients indicated that this encouragement and reinforcement of skills increased their resiliency and helped them get through difficult times.

### Theme 4: Peer provider roles and experiences specific to young adults, gender, and racial/ethnic minority groups

In the fourth theme identified in the analysis, clients reported benefits of their relationships with peer providers tied to “TAY-specific age and gender concordance” and “racial/ethnic or culture-specific peer provider roles”, which focused mainly on shared Latino identity. Relevant quotes for this theme are provided in Table 4.

**Table 4.** Peer Provider Roles & Experiences Specific to Young Adults, Gender, & Racial/Ethnic Minority Groups

Subtheme	Exemplary Quotes
**TAY-specific age & gender concordance**	**AGE** *It does matter to have someone who is a little older than you and has more experience in life because you’re going to relate to someone who’s been through your experiences and you can see that like, right now I’m not functioning but I can be them where they’re full-on adult and they’re functioning and they’re doing normal adult things even with all the symptoms that they experience that are like mine.* (Female 1, Group C) *Sometimes there’s things going on in your life that you want to get advice from older people, so it would be better to speak for someone who’s older than you vs. you’re going through something for the first time in your life and you want to get the experience of someone younger who just went through that.* (Female 1, Group A) *I would prefer somebody about my age for advice, maybe a little older but not too old. Because when they’re way older than me, they look down on my intelligence.* (Male 2, Group A) **GENDER** *I don’t think it matters about the gender, like we were talking about before. I have a male peer support and a female therapist and we both get along the same way so I don’t think it matters.* (Male 1, Group C) *Well, mental illness is not exactly easier being a woman so to actually be paired up with another woman who would experience the same, who pretty much understands that mood swings can be twice as bad, and certain symptoms can act up twice as bad during certain times of the month, that's really helpful.* (Female 1, Group C) **HOW TO DO THINGS AS AN ADULT** *We all know being an adult is fucking boring. Sometimes you have to do things you don’t like, like signing up for Medi-Cal, doing those things, getting insurance, all those things. That’s how [peers] help you. Just the essentials you feel like you need to learn, they teach you that, they let you know that this is what adults do.* (Male 3, Group C) *I don't really see myself relating with someone that’s like, close to my age like ‘okay you’re 2 years or 5 years ahead of me, buddy, like, oh well.’ But older people, I used to have a peer support where she was older and, she was, I don’t know, it was just fun to hang out with her, and the way she'll think differently, and I’m like ‘okay!’, like she’ll teach me like whenever you fill out forms, when you turn in forms, you always have to put out the date you filled out on top in the corner so that way you keep it filed, so you know, things that old people will do [laughter]. No offense, but you learn different things and you start seeing things in a different view as well, because you know every age has a different perspective in life.* (Male 3, Group C)
**Racial/ Ethnic or Culture-Specific Peer Provider Roles**	**DIVERSE ENVIRONMENT** ***Facilitator:*** *What about race or ethnicity? Is it important to have the same race as your peer or it doesn’t matter?* ***Male 1:*** *No, it doesn’t matter.* ***[group agreement]*** ***Female 2:*** *But I think it should be also important that it should be a mixed environment of ethnicity because you can kind of come and feel out of place when it's not diverse.* (Multiple Participants, Group A) **SHARED LATINO IDENTITY** ***Male 3:*** *And also the good thing about it is too is like how you're focusing on colored communities which is a great think because in the colored communities, mental health is not a thing, everyone knows it at this table, you know, our parents have always told us, if they can't see it, it doesn't exist. We all heard that at one point. So even our own, so with relating to someone, [my peer provider] is Latina and you know I'm Mexican so it's with her I could just talk to her too in Spanish and finally someone… because before when I was younger I used to, like I’ve been in therapy since I was six, when I was younger I would talk to the therapist and they would just be English speakers and I'm like okay, great I can't really reach you.* ***Facilitator:*** *You felt like that side of your life wasn't addressed?* ***Male 3:*** *Yeah because mostly our impact comes from our family’s descent, the way they perceive the mental health, the way they received it all to them it doesn't exist, like I said. It doesn't exist to some of them but it's just a whole new thing to them. And it's good to have someone like peer support who, on the other side went through the experience too and they had the same experience like they have the family as well. [My peer provider] said, ‘my family is the same way, we don’t, they didn't believe in it until things started happening.’* (Male 3, Group C) *Not even that, sometimes when you're just in the moment you just start speaking Spanglish and then you just start mixing up words and everything, it’s just a whole hot mess but at least they get it. Because with my therapist, sometimes I’ll start speaking Spanish because when I get pissed I just start speaking in Spanish because that's the first language I learned. So after that, my therapist is like ‘woah, woah, what does that mean’ and I’m like ‘forget it, you’re not [my peer provider], like you’re not her,’ you know but I’ll translate it, but with [my peer provider] I don't have to, we can just talk and then it’s just whatever. But it's helpful, it is helpful because if you know, sometimes when I talk about my family, and then she’s like ‘oohhh well this and this,’ you know when it’s always about family, we’ll talk about it all in Spanish for some reason. And then I’m like [to my therapist?] ‘we’re talking about our family here, and it’s all in Spanish right now, because I don’t want to translate what my mom will say or what my parents will say and I'm like you know what, I’m just going to tell you what she said in Spanish to me and that's how it will go off. So it was helpful.* (Male 3, Group C)

#### TAY-specific age and gender concordance

Several TAY clients had an opinion about whether or not peer providers should be similar in age or gender. Of these, most indicated or agreed that they would prefer that peer providers be slightly older than them and with more life experience in order to act as role models or mentors, but not substantially older as they might not relate to their experiences. Several participants also described peers assisting with tasks and skills associated with navigating the transition to adulthood, such as filling out paperwork or getting health insurance. Another client indicated it was helpful to have someone to talk to who had experienced mental illness during the same developmental period (i.e. late adolescence, early adulthood) and is now in recovery. Although most participants indicated that peers’ gender did not matter to them, one female participant reflected on how she appreciated having a female peer who could relate to specific challenges due to the intersection of mental health and female sexual and reproductive health.

#### Race/ethnicity or culture-specific peer provider roles

There was a diversity of opinion regarding the importance of racial and ethnic concordance between clients and peer providers. Some clients did not feel that that racial and ethnic concurrence with a peer provider was important, whereas other clients felt that racial and ethnic concordance between peers and providers was important. Specifically, several Latino clients described how they appreciated having a peer provider who spoke Spanish if they spoke Spanish; they also felt that peer providers with the same cultural background could relate specifically to culture-related struggles with family members regarding mental health. There was no explicit discussion about the experiences or preferences of African-American clients regarding peer providers.

## Discussion

This paper describes TAY clients’ perspectives regarding the roles of peer providers as they engage with mental health services. Our findings highlight the importance of shared lived experience, specifically with mental health challenges, as key to building connection and trust in client–peer provider relationships. Social support, in the form of instrumental and informational support to address basic needs and immediate goals, and emotional support, including having someone to talk to or to provide friendly encouragement, also contributed to building client–peer relationships.^36^ Clients’ characterizations of the expansive nature of the social support offered by peer providers suggest that it is an important component in their engagement with mental health care and recovery process.

Peer providers who can be seen as recovery role models and who act examples of successful mental health recovery and can serve as mentors or educators, may be especially important for TAY. In the present study, these factors contributed to the formation of a trusting relationship and increased rapport among clients and their peer providers. Role modelling and mentorship is one of the mechanisms by which peer providers are thought to improve clients’ self-efficacy and sense of empowerment, including overcoming previous negative experiences with mental health services,^37^ and instilling optimism for the future.^24,38^ This approach is illustrated by recently developed “Just Do You” program, which seeks to increase engagement in mental health services by pairing youth with adults who are older by at least a decade and have successfully navigated their own mental health challenges.^39–41^ In addition, the Cornerstone NYC program for TAY living with SMI integrated supplemented individual services with peer-led groups with the goal of creating a supportive mentoring environment in a group-based setting. In Cornerstone NYC, the peer-led groups implemented a structured curriculum in order to build TAYs’ knowledge and skills.^42^ Preliminary data suggest that this mentoring strategy was both feasible and acceptable to TAY and pilot data indicate that favourable results for depression status, symptoms, recovery, and stigma were observed among a pilot sample.^43^ Finally, a recent review of youth peers providers by Gopalan et al. reported that youth peers engage in important group-based roles in some interventions, such as group management, educator, and culture broker, among others.^44^ While the research based on the effectiveness of peers in providing group services in transition age youth mental health remains unclear, it is clear that some youth prefer peer support groups and the field can benefit from further research to deepen our understanding of the ways in which they contribute to the recovery process.

Clients identified the provision of a diverse array services and tangible resources to as a way that peers linked and engaged them with mental health services. This finding is consistent with our quantitative findings of increased service use among clients in programs where peers were engaged in diverse service roles.^26,27^ Clients recognized that peer providers helped them to navigate mental health services by reducing barriers to access, collaborating with other mental health team members to address client needs, and imparting specific informational support to help clients navigate mental health services. Other studies have similarly placed peers in the role of case service navigator.^10,24,44^ A recent review of mental health service navigation programs identified peer providers as the most frequently utilized team member for service navigation tasks.^45^ TAY may be especially in need of mental health service navigation as they are learning to navigate multiple systems as adults.^2,46^ Rooted in their lived experience, peer providers are often able to give practical and credible insights into navigating mental health systems.^10,47^ Exploring the ways in which peers providers are trained to engage in navigation activities is important and may help to create best practices for TAY-serving peer providers.

Spanish-speaking Latino TAY clients appreciated having a peer provider who spoke Spanish and could relate to the specific challenges associated with culturally specific family perceptions and stigma about mental health, as has been observed elsewhere.^48^ This finding is consistent with our previous quantitative findings showing that the availability of Latino peer providers was associated with greater engagement in outpatient mental health services among Latino TAY.^27^ Other recent studies have highlighted the role of cultural and family stigma as important barriers to mental health services among Latinos, the benefits of provider concordance among Latinos in terms of patient satisfaction and engagement in care, and the potential role of peer navigators to better support Latinos with SMI accessing both mental and physical health services.^49–51^

### Limitations

This analysis had some limitations. Providers led participant recruitment, which may have increased the risk of bias if only “model” clients experiencing favourable interactions with the program were recruited. One focus group was impacted by non-attendance of other scheduled participants, thus resulting in an individual interview. Additional recruitment activities were not scheduled due to saturation of themes within the existing sample. Female clients’ perspectives may have been under-represented. Although the focus groups included TAY of diverse racial/ethnic backgrounds, racial/ethnic or age data were not collected, limiting the investigators from assessing variations in experiences by age or racial/ethnic minority subgroups.

## Implications For Behavioural Health Interventions

These findings provide a nuanced understanding of how TAY clients relate to and benefit from peer providers’ support. Gale and colleagues recently coined the concept of “Synthetic Social Support” (SSS) in the context of a randomized clinical trial using Pregnancy Outreach Workers to increase use of prenatal care and reduce maternal depression.^52^ SSS was characterized as a time-limited, commissioned service that is designed to imitate the spontaneous social support provided by an individual’s close contacts; lay staff are held accountable for the relationship, while clients’ social networks are engaged in order to enhance within-network supports.^52^ The investigators found beneficial effects in terms of reduced rates of depression among women with two or more social risk factors. However, the intervention was constrained by its time-limited nature and lack of components designed to overcome structural inequalities that result in disparities.^52^

The work of peer providers, as described by TAY clients, shares many of the traits of SSS, such as commissioned social support characterized by accountability, but it may be time-limited due to programmatic constraints (e.g. age requirements). In this study, peer support as described by TAY clients was largely unstructured. Therefore, it is important to understand whether standardization of peer support services with inherent flexibility to meet clients’ emerging needs may have a greater or more consistent impact on client outcomes as compared to less structured peer support. For example, a recent observational pilot study employed a community worker to serve as a Service Navigator to support TAY probationers, many of whom had mental health challenges.^53^ Navigators used a structured intake to identify TAY clients’ health, social, economic, and resource supports needed, yet, navigators had flexibility to carry out their work in the office or community settings and to pivot to address emergent needs.^53^ Testing structured, peer support-focused interventions may shed light on effective and scalable strategies that can meet diverse TAYs’ needs. Additional work will be needed to determine in which ways such interventions may need to be tailored or adapted to the unique conditions of community settings or TAY subgroups’ needs.

## References

[1] Cusick GR, Havlicek JR, Courtney ME (2012). Risk for arrest: The role of social bonds in protecting foster youth making the transition to adulthood. American Journal of Orthopsychiatry..

[2] Davis M (2003). Addressing the needs of youth in transition to adulthood. Administration and Policy in Mental Health..

[3] Munson MR, McMillen JC (2009). Natural mentoring and psychosocial outcomes among older youth transitioning from foster care. Children and Youth Services Review..

[4] Vostanis P (2005). Patients as parents and young people approaching adulthood: How should we manage the interface between mental health services for young people and adults?. Current Opinion in Psychiatry..

[5] Bonnie RJ, Bonnie R, Stroud C, Breiner H (2015). *Investing in the health and well-being of young adults*. Committee on Improving the Health, Safety, and Well-Being of Young Adults; Board on Children, Youth, and Families; Institute of Medicine; National Research Council.

[6] Substance Abuse and Mental Health Services Administration. Key substance use and mental health indicators in the United States: Results from the 2019 National Survey on Drug Use and Health. HHS publication no. PEP20-07-01-001. Rockville, MD: Center for Behavioral Health Statistics and Quality, 2020: Available online at: https://www.samhsa.gov/data/sites/default/files/reports/rpt29393/2019NSDUHFFRPDFWHTML/2019NSDUHFFR090120.htm. Accessed 1 February, 2022.

[7] Substance Abuse and Mental Health Services Administration. Racial/ ethnic differences in mental health service use among adults. HHS Publication no. SNA-15-4906. Rockville, MD. February, 2015. Available online at: https://www.samhsa.gov/data/sites/default/files/MHServicesUseAmongAdults/MHServicesUseAmongAdults.pdf. Accessed 1 February, 2022.

[8] House JS. Barriers To Work Stress: I. Social Support. In: Gentry W, Benson H, de Wolff C (Eds.). *Behavioral Medicine: Work, Stress and Health.* NATO Science Series D: (closed) (Behavioural and Social Sciences), vol 19.1 ed. Dordrecht, Netherlands: Springer; 1985:157-180. 10.1007/978-94-009-5179-2_8

[9] Weiss RS, Rubin Z (1974). The provisions of social relationships. *Doing Unto Others*.

[10] Gillard S, Gibson S, Holley J (2015). Developing a change model for peer worker interventions in mental health services: A qualitative research study. Epidemiology and Psychiatric Sciences..

[11] Solomon P (2004). Peer support/peer provided services underlying processes, benefits, and critical ingredients. Psychiatric Rehabilitation Journal..

[12] Bandura A (1977). *Social Learning Theory*.

[13] Festinger L (1954). A theory of social comparison processes. Human Relations..

[14] Alegría M, Chatterji P, Wells K (2008). Disparity in depression treatment among racial and ethnic minority populations in the united states. Psychiatric Services..

[15] Stratford AC, Halpin M, Phillips K (2017). The growth of peer support: An international charter. Journal of Mental Health..

[16] Lloyd-Evans B, Mayo-Wilson E, Harrison B (2014). A systematic review and meta-analysis of randomised controlled trials of peer support for people with severe mental illness. BMC Psychiatry..

[17] Davidson L, Bellamy C, Guy K (2012). Peer support among persons with severe mental illnesses: A review of evidence and experience. World Psychiatry..

[18] Lloyd-Evans B, Mayo-Wilson E, Harrison B (2014). A systematic review and meta-analysis of randomised controlled trials of peer support for people with severe mental illness. BMC Psychiatry..

[19] Reif S, Braude L, Lyman DR (2014). Peer recovery support for individuals with substance use disorders: Assessing the evidence. Psychiatric Services..

[20] Jones N, Teague GB, Wolf J (2020). Organizational climate and support among peer specialists working in peer-run, hybrid and conventional mental health settings. Administration and Policy in Mental Health..

[21] Salzer MS, Schwenk E, Brusilovskiy E (2010). Certified peer specialist roles and activities: Results from a national survey. Psychiatric Services..

[22] Chinman M, McCarthy S, Mitchell-Miland C (2016). Early stages of development of a peer specialist fidelity measure. Psychiatric Rehabilitation Journal..

[23] King AJ, Simmons MB (2018). A systematic review of the attributes and outcomes of peer work and guidelines for reporting studies of peer interventions. Psychiatric Services..

[24] White S, Foster R, Marks J (2020). The effectiveness of one-to-one peer support in mental health services: A systematic review and meta-analysis. BMC Psychiatry..

[25] Mandarino K (2014). Transitional-age youths: Barriers to accessing adult mental health services and the changing definition of adolescence. Journal of Human Behavior in the Social Environment..

[26] Ojeda VD, Jones N, Munson MR (2021). Roles of peer specialists and use of mental health services among youth with serious mental illness. Early Intervention in Psychiatry..

[27] Ojeda VD, Munson MR, Jones N (2020). The availability of peer support and disparities in outpatient mental health service use among minority youth with serious mental illness. Administration and Policy in Mental Health and Mental Health Services Research..

[28] Glaser BG, Strauss AL (2017). *Discovery of Grounded Theory: Strategies for Qualitative Research*.

[29] Clarke V, Braun V (2014). Thematic Analysis. *Encyclopedia of Critical Psychology*.

[30] Saldaña J (2015). *The Coding Manual For Qualitative Researchers*.

[31] O’Connor C, Joffe H (2020). Intercoder reliability in qualitative research: Debates and practical guidelines. International Journal of Qualitative Methods..

[32] Roberts K, Dowell A, Nie J-B (2019). Attempting rigour and replicability in thematic analysis of qualitative research data; a case study of codebook development. BMC Medical Research Methodology..

[33] Landis JR, Koch GG (1977). The measurement of observer agreement for categorical data. Biometrics..

[34] Scott C, Medaugh M, Matthes J, Davis C, Potter R (2017). Axial coding. *The International Encyclopedia of Communication Research Methods*.

[35] Kendall J (1999). Axial coding and the grounded theory controversy. Western Journal of Nursing Research..

[36] Williams P, Barclay L, Schmied V (2004). Defining social support in context: A necessary step in improving research, intervention, and practice. Qualitative Health Research..

[37] McLeod B, Meyer D, Murray G (2019). Contact with recovered peers: Buffering disempowering service experiences and promoting personal recovery in serious mental illness. British Psychiatry Journal Open..

[38] Barker SL, Maguire N (2017). Experts by experience: Peer support and its use with the homeless. Community Mental Health Journal..

[39] Munson MR, Cole A, Jaccard JJ (2016). Just do you: An engagement intervention for young adults utilizing recovery role models. Journal of Behavioral Health Services and Research..

[40] Munson MR, Jaccard J, Smalling SE (2012). Static, dynamic, integrated, and contextualized: A framework for understanding mental health service utilization among young adults. Social Science and Medicine..

[41] Munson MR, Jaccard JJ, Scott LD (2020). Engagement intervention versus treatment as usual for young adults with serious mental illness: A randomized pilot trial. Pilot and Feasibility Studies..

[42] Munson MR, Cole A, Stanhope V (2016). Cornerstone program for transition-age youth with serious mental illness: Study protocol for a randomized controlled trial. Trials..

[43] Cole A, Munson M, Ben-David S, et al. Feasibility, acceptability, and preliminary impact of the cornerstone mentoring program [abstract]. In: Proceedings from the 10th Annual Conference on the Science of Dissemination and Implementation. *Implementation Science.* 2018;13:1-67. Abstract S18. 10.1186/s13012-018-0728-7

[44] Gopalan G, Lee SJ, Harris R (2017). Utilization of peers in services for youth with emotional and behavioral challenges: A scoping review. Journal of Adolescence..

[45] Waid J, Halpin K, Donaldson R (2021). Mental health service navigation: A scoping review of programmatic features and research evidence. Social Work in Mental Health..

[46] Osgood DW, Foster EM, Courtney ME (2010). Vulnerable populations and the transition to adulthood. Future Child..

[47] Proudfoot JG, Jayawant A, Whitton AE (2012). Mechanisms underpinning effective peer support: A qualitative analysis of interactions between expert peers and patients newly-diagnosed with bipolar disorder. BMC Psychiatry..

[48] Misra S, Jackson VW, Chong J (2021). Systematic review of cultural aspects of stigma and mental illness among racial and ethnic minority groups in the united states: Implications for interventions. American Journal of Community Psychology..

[49] Alegría M, Roter DL, Valentine A (2013). Patient–clinician ethnic concordance and communication in mental health intake visits. Patient Education and Counseling..

[50] Rivera-Segarra E, Varas-Díaz N, Santos-Figueroa A (2019). “That’s all fake”: Health professionals stigma and physical healthcare of people living with serious mental illness. Plos One..

[51] Corrigan PW, Torres A, Lara JL (2017). The healthcare needs of latinos with serious mental illness and the potential of peer navigators. Administration in Policy in Mental Health and Mental Health Services Research..

[52] Gale NK, Kenyon S, MacArthur C (2018). Synthetic social support: Theorizing lay health worker interventions. Social Science and Medicine..

[53] Ojeda VD, Berliant E, Parker T, et al. Overview of a pilot health-focused reentry program for racial/ethnic minority probationers ages 18 to 26 in southern california. *International Journal of Offender Therapy and Comparative Criminology.* 2021: May:1-24. 10.1177/0306624X211013739. 10.1177/0306624X211013739 PMC12702459 33980068

